# Integrated metabolomics and transcriptomics to reveal biomarkers and mitochondrial metabolic dysregulation of premature ovarian insufficiency

**DOI:** 10.3389/fendo.2023.1280248

**Published:** 2023-12-21

**Authors:** Zhaoyang Yu, Weilong Peng, Feiwen Li, Xiaoqian Fu, Jiajia Wang, Hongfan Ding, Mujun Li, Huimei Wu

**Affiliations:** ^1^ Guangxi Medical University, Nanning, China; ^2^ School of Computer Science and Cyber Engineering, Guangzhou University, Guangzhou, China; ^3^ Guangxi Reproductive Medical Center, The First Affiliated Hospital of Guangxi Medical University, Nanning, China; ^4^ Department of Obstetrics and Gynecology, Affiliated Hospital of Youjiang Medical University for Nationalities, Baise, China

**Keywords:** premature ovarian insufficiency, metabolomics, transcriptomics, biomarkers, machine learning, mitochondrial dysfunction

## Abstract

**Background:**

The metabolic characteristics of premature ovarian insufficiency (POI), a reproductive endocrine disease characterized by abnormal sex hormone metabolism and follicle depletion, remain unclear. Metabolomics is a powerful tool for exploring disease phenotypes and biomarkers. This study aims to identify metabolic markers and construct diagnostic models, and elucidate the underlying pathological mechanisms for POI.

**Methods:**

Non-targeted metabolomics was utilized to characterize the plasma metabolic profile of 40 patients. The metabolic markers were identified through bioinformatics and machine learning, and constructed an optimal diagnostic model by classified multi-model analysis. Enzyme-linked immunosorbent assay (ELISA) was used to verify antioxidant indexes, mitochondrial enzyme complexes, and ATP levels. Finally, integrated transcriptomics and metabolomics were used to reveal the dysregulated pathways and molecular regulatory mechanisms of POI.

**Results:**

The study identified eight metabolic markers significantly correlated with ovarian reserve function. The XGBoost diagnostic model was developed based on six machine learning models, demonstrating its robust diagnostic performance and clinical applicability through the evaluation of receiver operating characteristic (ROC) curve, decision curve analysis (DCA), calibration curve, and precise recall (PR) curve. Multi-omics analysis showed that mitochondrial respiratory chain electron carrier (CoQ10) and enzyme complex subunits were down-regulated in POI. ELISA validation revealed an elevation in oxidative stress markers and a reduction in the activities of antioxidant enzymes, CoQ10, and mitochondrial enzyme complexes in POI.

**Conclusion:**

Our findings highlight that mitochondrial dysfunction and energy metabolism disorders are closely related to the pathogenesis of POI. The identification of metabolic markers and predictive models holds significant implications for the diagnosis, treatment, and monitoring of POI.

## 1 Introduction

Premature ovarian insufficiency (POI) refers to the failure of ovarian reproductive and endocrine function in women before the age of 40 (1). The most common clinical manifestations of POI are menstrual disorders and infertility. Long-term complications include cardiovascular disease, osteoporosis, and neurodegenerative diseases (1), which are partly attributed to the lack of protective effects of estrogen. The etiology of POI is highly heterogeneous, encompassing genetic, immune-mediated, infectious and iatrogenic factors, however, 60% of the etiology remains unknown (2). Decreased ovarian reserve (DOR) is the initial stage of ovarian dysfunction, which can develop into POI if it persists, and premature ovarian failure (POF) is the final stage (3). Hormone replacement therapy (HRT) is currently the most commonly utilized treatment for POI; however, prolonged HRT use may elevate cancer risk (4). Given the endocrine dyscrasia, absence of early diagnostic indicators, and incurable nature of POI, it is imperative to identify novel effective markers and therapeutic targets for POI diagnosis, treatment, and monitoring.

Metabolomics utilizes advanced chemical analysis techniques to characterize metabolites in cells, tissues, and body fluids in a high-throughput manner, rendering it a powerful tool for studying disease phenotypes (5). As downstream products of molecular regulation, metabolites can directly reveal the functions of upstream genes and proteins (5). Metabolomics has emerged as a promising tool for predicting and improving reproductive outcomes through biomarker identification (6). POI is characterized by high gonadotropins and low estrogen levels, which are closely related to metabolic disorders. Previous studies demonstrated that POI is associated with glucose and lipid metabolism disorders and an increased risk of metabolic syndrome (7). Metabolic dysregulation may underlie the long-term complications of POI; therefore, deciphering the molecular network of POI may provide meaningful evidence for its pathogenesis and the identification of key biomarkers.

This study aimed to utilize ultra-high throughput liquid chromatography-mass spectrometry (LC-MS) non-target metabolomics to analyze plasma metabolites of POI. By combining bioinformatics, weighted gene co-expression network analysis (WGCNA), and Gaussian naive Bayes (GNB) algorithms, we identified metabolic markers and pathways and constructed an optimal diagnostic model through machine learning (ML). We also integrated our previous full-length transcriptome data to elucidate the molecular regulatory mechanism underlying POI and provided novel insights into its pathogenesis, clinical diagnosis, and treatment.

## 2 Materials and methods

### 2.1 Inclusion and exclusion criteria for participants

This study recruited 40 participants (20 POI and 20 control patients) from the Reproductive Center of the First Affiliated Hospital of Guangxi Medical University. Inclusion criteria for POI included: (i) age < 40 years, (ii) oligomenorrhea or amenorrhea for at least 4 months, and (iii) two basal follicle stimulating hormone (FSH) levels > 25 IU/L with an interval > 4 weeks. The control group had to meet the following inclusion criteria: (i) matching age and weight with the POI group, (ii) infertility caused by male or tubal factors, and (iii) regular menstrual cycles with normal basal sex hormones. Exclusion criteria common to all participants included: (i) the presence of other endocrine or autoimmune conditions, such as hyperthyroidism, thyroiditis, and polycystic ovary syndrome (PCOS); (ii) a history of pelvic surgery and chemoradiotherapy; (iii) the use of hormones or drugs affecting endocrine metabolism within three months before blood collection; (iv) severe systemic illness; and (v) an abnormal chromosome karyotype. The clinical data of all participants were collected, including age, body mass index (BMI), anti-Mullerian hormone (AMH), FSH, luteinizing hormone (LH), estradiol (E2), progesterone (P), and antral follicle count (AFC). Informed consent was obtained from all participants and this study was approved by the Ethics Committee of the First Affiliated Hospital of Guangxi Medical University (NO.2021KY-E-249).

### 2.2 Collection and pre-processing of peripheral blood samples

Peripheral blood (3 ml) was collected in ethylenediamine tetraacetic acid-anticoagulated tubes on days 2–4 of the menstrual cycle, centrifuged at 3000 rpm for 10 minutes at 4°C within 1 hour, and stored at -80°C until analyzed by ultrahigh-performance liquid chromatography-tandem mass spectrometry (UHPLC-MS/MS). After thawing at 4°C, a sample of 100 μL was added to an extraction solution containing internal standards (methanol: acetonitrile = 1:1). The mixture was vortexed for 30 seconds, placed in an ice water bath for 10 minutes, and centrifuged at 12000 rpm for 15 minutes at 4°C. After drying in an Eppendorf tube, the resulting mixture was redissolved with 160 μL of extract solution (acetonitrile:water = 1:1) and centrifuged again at the same conditions. Finally, a supernatant of 120 μL was transferred into a 2 mL injection vial. To assess sample preparation repeatability and instrument stability, equal amounts of each sample were mixed and used as quality control (QC).

The LC-MS system used for metabolomics analysis consisted of an Acquity I-Class PLUS UHPLC (Waters, Ireland) in tandem with an Xevo G2-XS QTOF high-resolution mass spectrometer (Waters, Ireland). Chromatographic separation was conducted on an Acquity UPLC HSS T3 column at a flow rate of 400 μl/min using a linear gradient of 15 min. The mobile phases A and B were composed of aqueous solution containing 0.1% formic acid and acetonitrile containing 0.1% formic acid, respectively. The mass spectrometer was operated in both positive and negative polarity modes, with the ion source parameters set as follows: capillary voltage of 2500V (in positive ion mode) or -2000V (in negative ion mode), cone voltage of 30V, ion source temperature of 100°C, desolvation temperature of 500°C, backblowing flow rate of 50L/h, and desolvation gas flow rate of 800L/h.

### 2.3 Data analysis

Statistical, bioinformatics, and ML analyses were performed. Data were analyzed using SPSS version 24.0 (IBM, Armonk, NY, USA). Normally distributed continuous variables were compared using Student’s t-test and presented as mean ± standard deviation. Non-normal distributions were compared using the Mann–Whitney U test and presented as median (interquartile range). Univariate (T test) and multivariate statistical analysis, including principal component analysis (PCA) and orthogonal partial least squares discriminant analysis (OPLS-DA), were employed to identify differentially expressed metabolites. PCA was primarily utilized for sample clustering trend determination and quality control assessment, with the first principal component PC1 and second principal component PC2 representing the contribution proportion of each sample to the observed differences. OPLS-DA was applied to compare dissimilarities between the two groups. The interpretability of the model for categorical variable Y was evaluated using R2Y, while predictability was assessed through Q2Y. Permutation tests were conducted to mitigate potential risks associated with overfitting. The criteria for identifying differentially expressed metabolites (DEMs) between both groups were VIP >1, *P <*0.05, and fold change (FC) ≥1.

**Figure 1:**
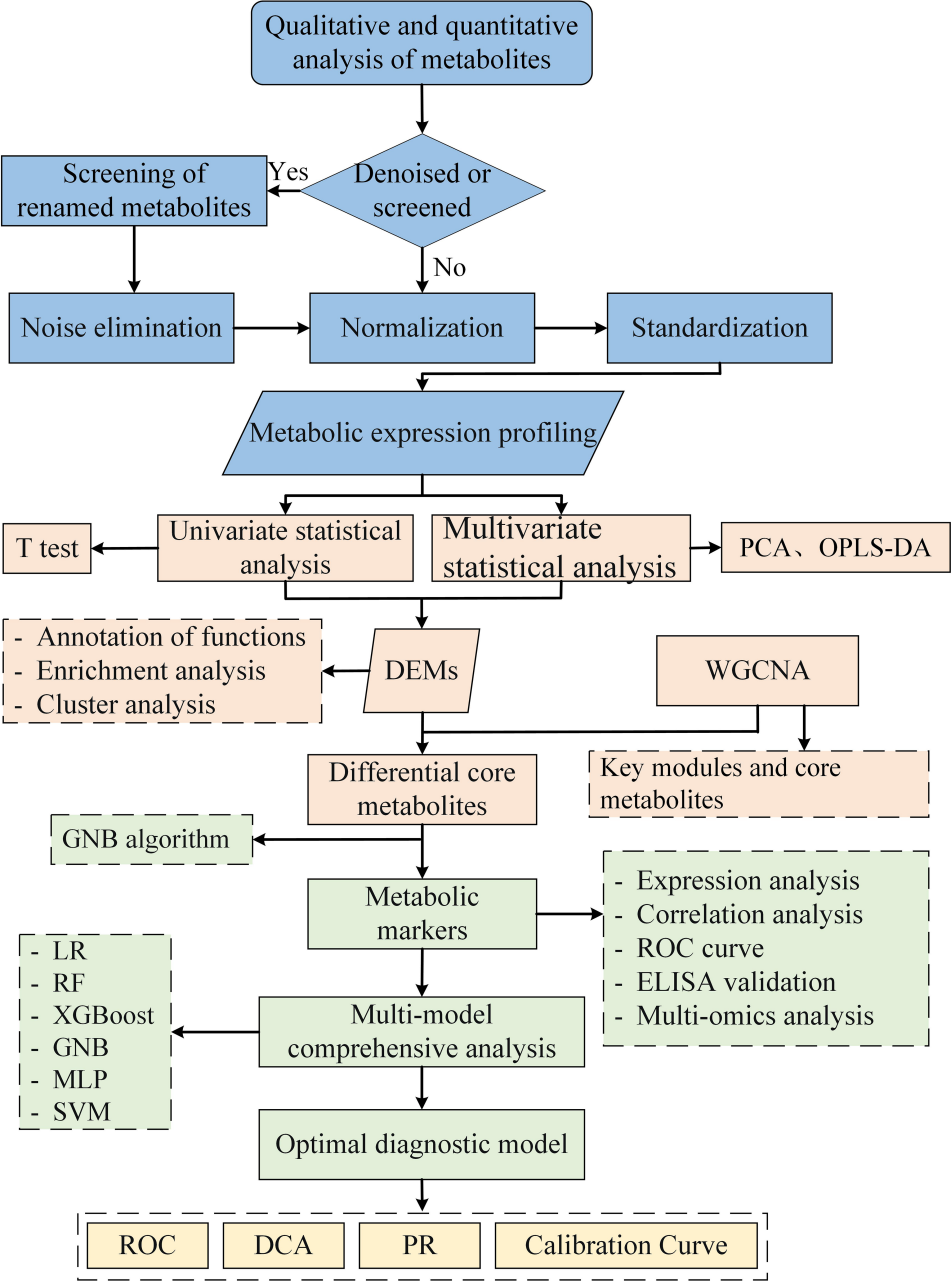
Flow chart of metabolomics analysis.

The Kyoto Encyclopedia of Genes and Genomes (KEGG) database was utilized for functional annotation and pathway enrichment analysis of metabolites, and Fisher’s exact test was employed to calculate the significance level of enriched pathways. Co-expressed modules of metabolic profiles were identified through WGCNA analysis, and key modules significantly associated with disease phenotypes were determined based on |r|≥0.5 and P < 0.05 thresholds. Within the metabolites of key module, further screening was conducted to identify core metabolites using criteria of |module member| > 0.8 and |gene significance| > 0.2. Finally, the top eight metabolites screened by weight importance index of GNB algorithm (Python sklearn 0.22.1) were identified as metabolic markers. The diagnostic potential of metabolic markers was evaluated by the area under the curve (AUC) of the receiver operating characteristic (ROC). Clinical correlation was analyzed using Pearson correlation. The analysis flow for this study is shown in **Figure 1**.

### 2.4 Construction and evaluation of diagnostic models

Based on the characteristic variables screened by GNB, a multimodel comprehensive analysis was performed to construct the optimal diagnostic model. Python (sklearn 0.22.1, xgboost 1.2.1) was used to build six prediction models: logistic regression (LR), extreme gradient boosting (XGBoost), random forest (RF), support vector machine (SVM), multilayer perceptron (MLP), and GNB. Each model’s predictive ability was evaluated using the AUC value under the ROC curve. The clinical applicability and diagnostic performance of each model were evaluated using decision curve analysis (DCA), calibration curve, and precision-recall (PR) curve.

### 2.5 Enzyme-linked immunosorbent assay of plasma

To evaluate the antioxidant capacity and mitochondrial function of POI, peripheral blood samples were obtained from 40 patients. The levels and activities of superoxide dismutase (SOD), glutathione peroxidase (GSH-PX), coenzyme Q10 (CoQ10), and mitochondrial enzyme complexes were quantified using ELISA kits (Jiangsu Meimian industrial Co., Ltd) in accordance with the manufacturer’s instructions.

### 2.6 Integrated transcriptome data revealed pathways of metabolic dysregulation

KEGG enrichment analysis were conducted to identify the dysregulated pathways of POI by integrating full-length transcriptome with metabolomics data. Additionally, regulatory roles of DEMs and DEGs in mitochondrial function and energy metabolism disorders were further analyzed. Our RNA sequencing dataset can be accessed at NCBI under bioproject (accession number: PRJNA964483).

## 3 Result

### 3.1 Clinical characteristics of the participants

The clinical features of the participants in both groups are presented in **Table 1**. No significant differences were observed between the two groups with respect to age, BMI, and serum P (*P* > 0.05). However, basal FSH and LH levels were significantly higher in POI patients compared to controls while AMH, E2 and AFC levels were significantly lower (*P* < 0.05). These findings suggest that women with POI exhibit typical characteristics of elevated gonadotropin levels and reduced estrogen production.

**Table 1:** Baseline data and clinical characteristics of the participants.

Parameter	Control (n=20)	POI (n=20)	*P*-value
Age (year)	33.40 ± 3.15	34.00 ± 2.90	0.535
BMI (kg/m^2^)	22.11 ± 0.46	22.58 ± 0.27	0.094
AMH (ng/mL)	3.62 ± 1.06	0.12 ± 0.12	<0.001^*^
FSH (mIU/mL)	5.35 ± 0.99	33.54 ± 8.12	<0.001^*^
LH (mIU/mL)	5.13 ± 0.63	18.95 ± 3.92	<0.001^*^
E2 (pg/mL)	45.23 ± 6.19	23.20 ± 4.91	<0.001^*^
P(nmol/L)	0.37 ± 0.09	0.33 ± 0.09	0.118
AFC (n)	14.25 ± 2.79	1.75 ± 1.02	<0.001^*^

“*” indicated P value<0.05.

### 3.2 Multivariate analysis of metabolites

**Figure 2:**
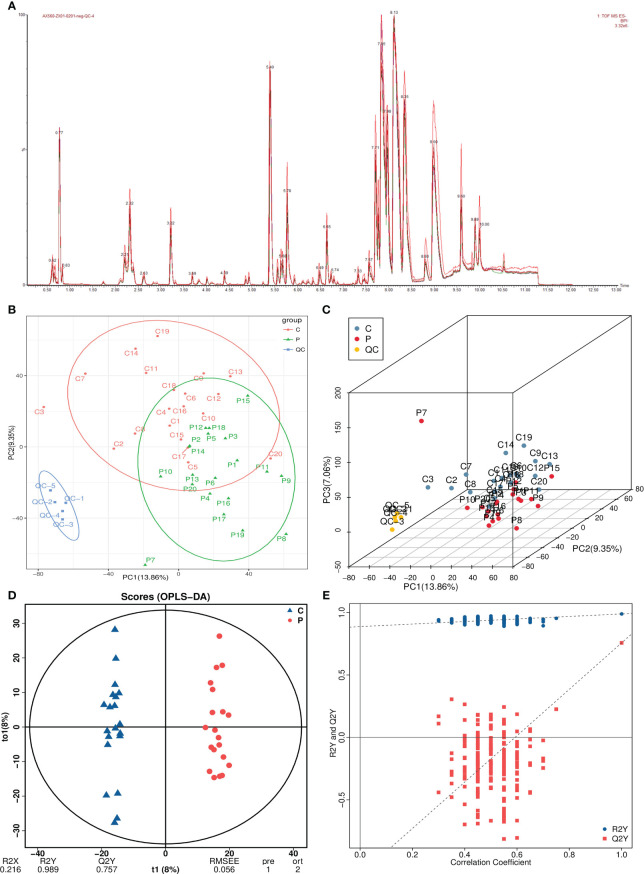
Multivariate statistical analysis of metabolomics. **(A)** Total ion current plots of metabolites. **(B, C)** PCA plots and three-dimensional plots of differential grouping and QC. **(D, E)** OPLS-DA score plot and permutation test.

A total of 9,474 peaks were detected in both positive and negative ion modes, which were annotated to 3,227 metabolites (**Figure 2A**). After noise removal, normalization and standardization procedures were applied, and the final dataset was obtained. The PCA score plot showed a clear separation between QC and study samples, indicating consistent processing and stable detection. While control group samples clustered at the top, POI group samples primarily grouped at the bottom. However, there was some overlap in the first and second principal components, suggesting differences, but not significant or absolute (**Figures 2B, C**).

Therefore, we used OPLS-DA analysis, a supervised dimensionality reduction method, to maximize the difference between both groups. In the OPLS-DA model, R2Y represents matrix interpretation probability, and Q2Y represents model predictability. Higher values of these parameters indicate greater reliability of our model. The analysis revealed significant metabolite alterations in both groups, with a distinct separation pattern observed (**Figure 2D**). The model exhibited high reliability (R2Y=0.989) and good predictive performance (Q2Y=0.757). The permutation test plot showed that the R2 value was higher than the Q2 value (blue dots above red dots), and the intercept of the Q2 regression line with the Y-axis was <0, indicating no overfitting in the OPLS-DA model (**Figure 2E**).

### 3.3 Identification of differential metabolites and pathways

**Figure 3:**
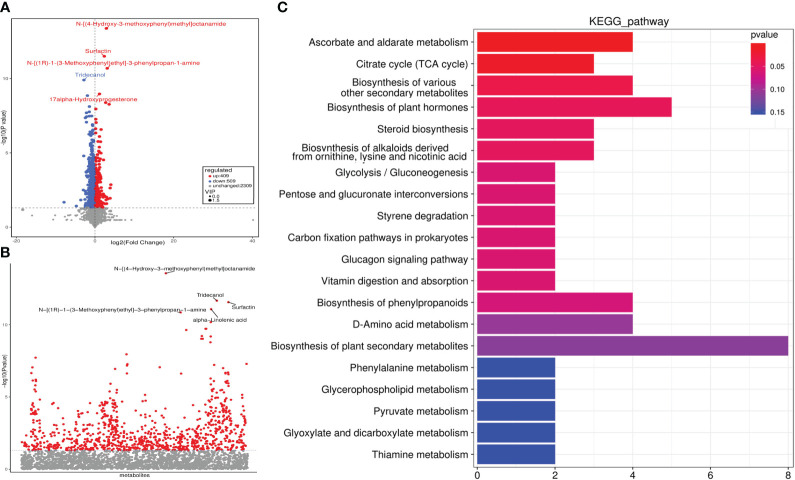
Identification of differential metabolites and KEGG enrichment analysis. **(A)** Volcano plot. **(B)** ANOVA scatter plot. **(C)** KEGG pathway enrichment analysis.

A total of 918 DEMs were identified, of which 409 were up-regulated and 509 were down-regulated in the POI group (**Supplementary Table 1**). The volcano plot and ANOVA scatter plot were used to visualize expression differences and significance of metabolites (**Figures 3A, B**). KEGG enrichment analysis showed that the top five pathways enriched for DEMs were ascorbic acid and aldarate metabolism, citric acid cycle (TCA cycle), biosynthesis of various other secondary metabolites, biosynthesis of plant hormones, and steroid biosynthesis (**Figure 3C**; **Supplementary Table 2**).

### 3.4 Identifying core metabolites by WGCNA

**Figure 4:**
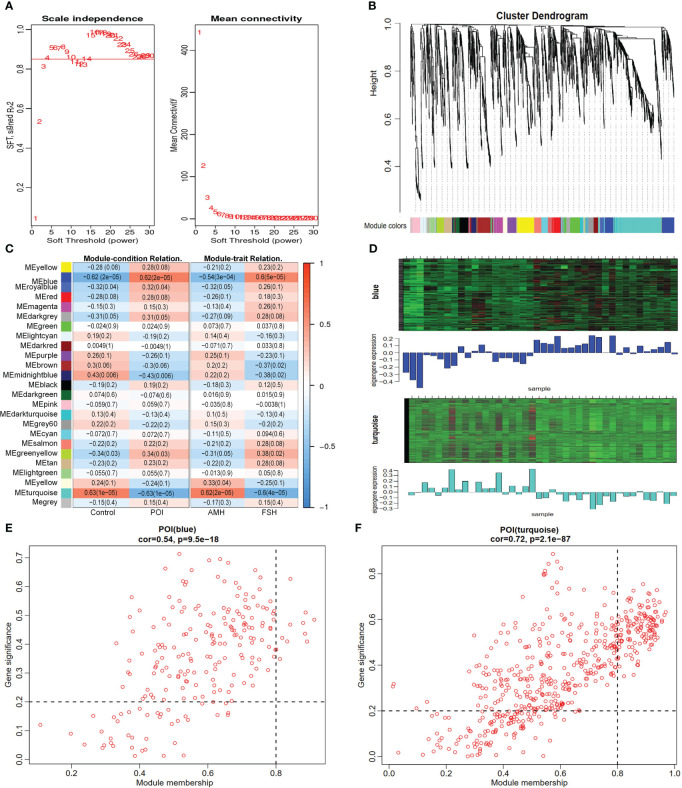
Identification of core metabolites by WGCNA. **(A)** identification of the optimal soft threshold. **(B)** classification of metabolite cluster modules. **(C)** correlation analysis of modules and traits. **(D)** Heat map of metabolites expression in key modules. **(E, F)** scatter plot of module attribution and metabolite significance.

WGCNA was conducted on the metabolic profiles to identify co-expression modules, core metabolites, and correlations between metabolites and specific phenotypes. The cluster tree of the sample system was constructed using the class average method, suggesting no deviated samples (**Supplementary Figure 1**). The optimal β value was determined through a soft thresholding approach, and a power value of 4 yielded a scale-free network fit index R^2^ >0.85 (**Figure 4A**). Twenty-five modules were identified and visualized using heat maps generated by dynamic shear mixing, with the gray module representing unassigned metabolites to any particular module (**Figure 4B**). Finally, two key modules (blue and turquoise modules), including 756 core metabolites, were identified by |r| ≥0.5 and *P* <0.05. The blue module exhibiting a significant positive correlation and the turquoise module displaying a significant negative correlation with POI (**Figure 4C**; **Supplementary Table 3**). Similarly, the blue module was significantly negatively correlated with AMH, and the turquoise module was significantly positively correlated with FSH. These core metabolites showed opposite expression trends in the blue and turquoise modules (**Figure 4D**). Within the metabolites of key module, a total of 152 core metabolites were identified as closely associated with key modules and disease phenotypes based on the criteria of |module membership|>0.8 and |gene significance|>0.2, (**Figures 4E, F**), warranting further investigation.

### 3.5 Screening feature variables using the GNB algorithm

**Figure 5:**
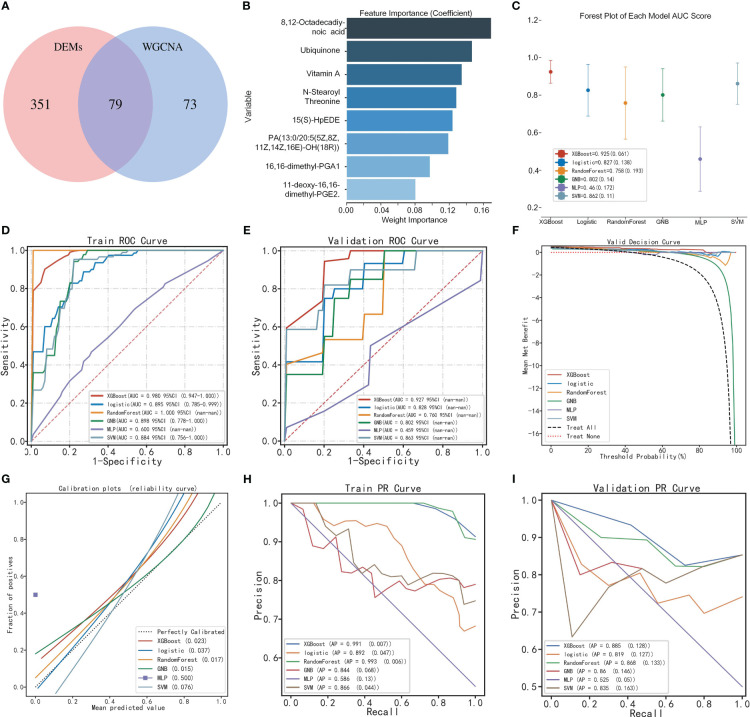
Construction of the diagnostic model by multi-model analysis. **(A)** Venn diagram of DEMs and core metabolites. **(B)** Identification of feature variables by GNB algorithm. **(C)** Forest plot of multi-model AUC scores. **(D, E)** ROC curve of multi-model based on training and validation sets. **(F)** Decision curve analysis. **(G)** Calibration curve analysis. **(H, I)** Precision-recall curve of multi-model based on training and validation sets.

**Figure 6:**
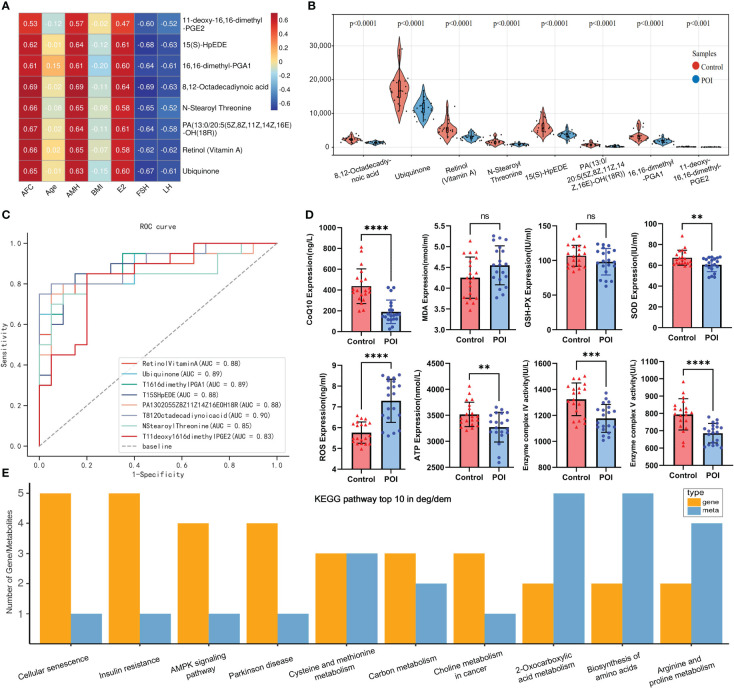
Metabolic marker analysis and multi-omics KEGG enrichment analysis. **(A)** Heat map of clinical relevance of metabolic markers. **(B)** Violin plot of metabolic markers expression in metabolic profile. **(C)** ROC curves of metabolic markers. **(D)** Plasma ELISA validation of CoQ10, MDA, GSH-PX, SOD, ROS, ATP, complex VI and V in both groups. ** indicated *P <*0.01 and **** indicated *P* < 0.0001. **(E)** top10 pathway was enriched by differential genes and metabolites.

The intersection of core and differential metabolites resulted in 79 differential core metabolites (**Figure 5A**; **Supplementary Table 4**). The GBN algorithm can effectively prevent overfitting by utilizing prior probability and assuming feature independence. Finally, the top eight characteristic variables screened using the GNB algorithm were defined as candidate metabolic markers, including: 8,12-Octadecadiynoic acid, Ubiquinone, Retinol (Vitamin A), N-Stearoyl Threonine, 15(S)-HpEDE, PA (13:0/20:5(5Z,8Z,11Z,14Z,16E)-OH (18R)), 16,16-dimethyl-PGA1, 11-deoxy-16,16-dimethyl-PGE2 (**Figure 5B**). The violin plot indicated a down-regulation of all metabolic markers in the POI group within the metabolic profile (**Figure 6B**). The clinical correlation analysis revealed significant positive correlations between metabolic markers and AFC, AMH, and E2 (r > 1, *P* < 0.05), as well as significant negative correlations with FSH and LH (r < 1, *P* < 0.05) (**Figure 6A**). Additionally, the ROC curve demonstrated that these metabolic markers had AUC values ranging from 0.83 to 0.9, indicating high diagnostic potential (**Figure 6C**; **Supplementary Table 5**).

### 3.6 Construction of the optimal diagnostic model using multi-model analysis

Based on the metabolic markers, six ML algorithms were used for classified multi-model comprehensive analysis to construct the optimal diagnostic model. The ROC curves demonstrated that the XGBoost model exhibited superior performance in discriminating POI from controls, with an AUC of 0.980 and 0.927 for the training and validation sets, respectively (**Figures 5D, E**). The forest plot further demonstrates that XGBoost exhibits a smaller standard deviation of AUC (SD=0.019), indicating a higher level of model stability (**Figure 5C**). However, the AUC value alone cannot fully evaluate the clinical utility of the model, and we conducted DCA, calibration curve and PR curve analysis. The DCA curves showed superior clinical applicability of both XGBoost and LR models (**Figure 5F**). Furthermore, calibration curves indicated that GNB (0.015), RF (0.017), and XGBoost (0.023) achieved better Brier scores with smaller differences between predicted and observed values, suggesting higher prediction accuracy (**Figure 5G**). The XGBoost and RF models showed superior AP values in PR curve within train and validation sets, resulting in higher precision and recall rates for more accurate identification of true positives (**Figures 5H, I**). A comprehensive analysis confirmed that the XGBoost model represents the optimal diagnostic tool in terms of discrimination, calibration, and clinical applicability.

### 3.7 Results of plasma ELISA

The ELISA results indicated that the levels of SOD, ATP and CoQ10 were significantly lower in the POI group compared to the control group. Similarly, the enzymatic activities of mitochondrial complexes IV and V were significantly lower in the POI group (*P* < 0.05). However, ROS levels were significantly higher in the POI group (*P* < 0.05). There was no significant difference in MDA and GSH-PX between both groups (*P* > 0.05). These findings further support the notion that oxidative stress and mitochondrial dysfunction may play an important role in POI pathogenesis (**Figure 6D**).

### 3.8 Integrated metabolomics and transcriptomics revealed dysregulated pathways and mitochondrial dysfunction

**Figure 7:**
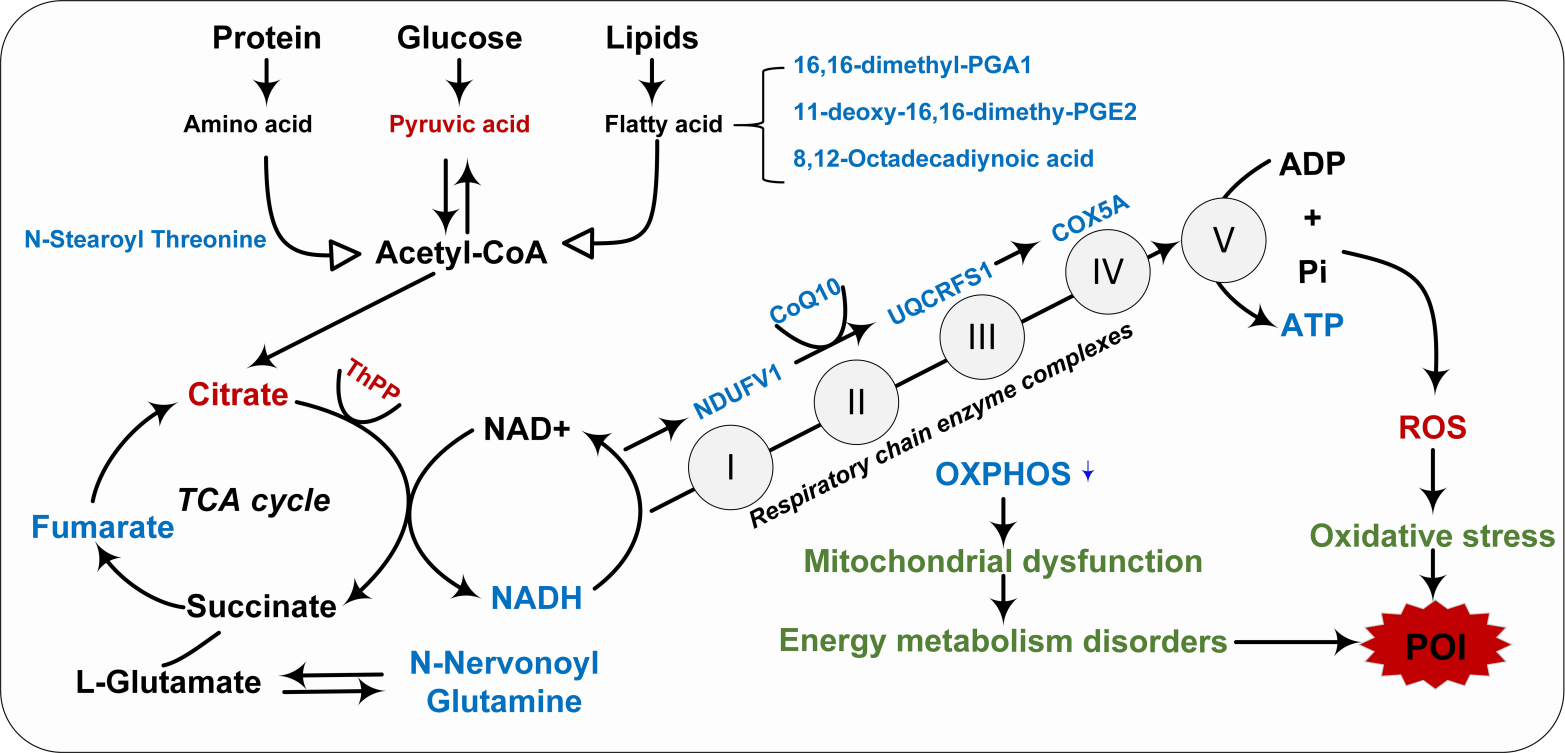
Integrated transcription and metabolomics revealed the pathological mechanism of impaired mitochondrial energy metabolism in POI. Red indicated upregulation, blue indicated downregulation, and green indicated pathological mechanisms.

KEGG enrichment analysis showed that pathways enriched by DEGs and DEMs included cell senescence, insulin resistance, AMPK signaling pathway, biosynthesis of amino acids, arginine and proline metabolism, and other pathways (**Figure 6E**). Our previous transcriptome study showed that the down-regulation of mitochondrial respiratory chain enzyme complex subunits play a crucial role in the pathogenesis of POI (**Supplementary Figure 2**) (8). The metabolic markers identified in this study were involved in antioxidant capacity and energy metabolism, among which CoQ10 as MRC electron carrier was downregulated in POI. Hence, it is plausible to hypothesize that the malfunction of mitochondrial respiratory chain electron carriers and enzyme complexes collectively results in an energy metabolism imbalance within the oxidative phosphorylation pathway, which may be intricately linked to the pathological mechanism of POI (**Figure 7**).

## 4 Discussion

POI is a reproductive endocrine disorder characterized by pathological sex hormone imbalances and follicular depletion. It is closely associated with metabolic disorders, which often underlie the pathogenesis of long-term complications in POI. However, the pathogenesis and metabolic changes underlying POI remain unclear. In this study, the plasma metabolites of POI were characterized using UHPLC-MS/MS, and 918 metabolites were identified (409 up-regulated and 509 down-regulated). Using multivariate statistical analysis, WGCNA, and ML, eight metabolite markers were identified, involved in unsaturated fatty acids (UFAs), antioxidants, and amino acids. Based on a comprehensive evaluation of AUC, DCA, calibration curve, and PR curve, the XGBoost model was identified as the optimal diagnostic tool. This finding provides promising prospects for individual prediction and clinical application, contributing to the improvement of the POI diagnostic system and offering valuable guidance for clinicians.

In this study, UFAs and their derivatives constituted the majority of metabolic markers. Some metabolomics studies have shown that POI is closely related to lipid metabolism disorders (9, 10). 15(S)-HpEDE and prostaglandins are both metabolites of arachidonic acid catalyzed by cyclooxygenase. Recent metabolomics revealed that 11,12-epoxyeicosatrienoic acid, 9(S)HPETE, and 20-hydroxyeicosatetraenoic acid were downregulated in ovarian tissue of POI mice. However, these metabolites returned to normal levels after mesenchymal stem cell treatment (10). These findings suggest that abnormal lipid metabolism in ovarian tissue closely related to POI (10). Another follicular fluid lipid metabolomics study also supported our results, showing that 15 differential lipid metabolites enriched in the tetraenoic acid metabolic pathway were down-regulated in DOR, including ±20-HDoHE, 12S-HHTrE, 8S, PGA1, and PGE2 (9). 11-deoxy-16,16-dimethyl-PGE2 is a derivative of prostaglandin E2 (PEG2), which serves as a crucial regulator in germ cell development during ovarian maturation (11). Moreover, PEG2 has been demonstrated to play pivotal roles in ovulation, fertilization, embryonic growth and early implantation associated with female reproduction (12). More evidence suggests that PEG2 mediates gonadotropin-stimulated cumulus expansion and oocyte maturation (13), and plays a key role in protecting oocytes from oxidative stress during this process (14). 8, 12-octadecadiynoic acid is an unsaturated fatty Acid. Studies have shown that 9, 12-octadecadiynoic acid positively regulates neuronal activity by affecting antioxidant genes (15), and has been identified as a potential marker for rheumatoid arthritis (16). In summary, fatty acids and their derivatives regulate follicular development, oocyte maturation and embryonic development by participating in energy metabolism as well as synthesizing precursors for steroid hormones and prostaglandins (17).

Phosphatidic acid (PA) is the primary product of lipolysis activated by phospholipase D. It plays a crucial role in regulating various biological processes, including cell growth, proliferation, reproduction, and signal pathway activation (18). PA (13:0/20:5(5Z,8Z,11Z,14Z,16E)-OH (18R)) indicates that the first and second fatty acid chains consist of 13 and 20 carbon atoms respectively with a hydroxyl group (OH) located at the 18th carbon atom. PA is closely related to mitochondrial function and morphology, playing a crucial role in mitochondrial membrane biogenesis, energy metabolism, and signal transduction pathways. Moreover, phospholipids regulate both membrane fluidity and permeability, which are key parameters for the survival of sperm, oocytes and embryos after cryopreservation (19, 20). An age-related lipid metabolomic showed that PA, phosphatidylinositol, and phosphatidylserine were significantly down-regulated in oocytes from aged-mice and H2O2-treated mice compared with young mice, suggesting that these phospholipids are essential for maintaining plasma membrane integrity and related to fertilization and developmental potential of oocytes (21). Similarly, PA levels were higher in the follicular fluid of pregnant women than in non-pregnant groups, indicating that these lipids are involved in steroidogenesis, cellular responses, signal transduction, cell cycle regulation and protein kinase C activation during pregnancy (22). Therefore, we speculated that PA could be involved in the pathophysiology of POI by affecting mitochondrial and oocyte membrane structure and function and activation of the PI3K/AKT/mTOR signaling pathway.

Retinol, also known as vitamin A (VA), is an antioxidant that plays a crucial role in reproduction. Previous studies have demonstrated the regulatory effects of VA on follicular development, oocyte maturation, ovarian steroid hormone production and luteal formation (23, 24). VA levels in follicular fluid are closely related to human oocyte quality, fertilization potential, and embryonic development (25), which benefit from the antioxidant properties of VA itself and the anti-apoptotic effect of enhanced transcription of other antioxidant enzymes, such as SOD and GSH-PX (23). Recent metabolomics studies have also shown that retinol and its metabolites are downregulated in follicular fluid and peripheral blood of women with DOR and POF (26, 27). Previous case-control studies have shown that retinol-binding protein 4 (RBP4) reflects blood retinol concentrations and is downregulated in DOR (28, 29). Overexpression of RBP4 upregulates FSH receptors of granulosa cells and thus improving ovarian response to hormones (30), which indirectly reflects the positive effect of VA on ovarian function. Interestingly, dextran sodium sulfate-induced colitis and intestinal flora dysbiosis in mice lead to impaired VA absorption and metabolism, affecting follicle development and steroid hormone secretion (31). This suggests that VA plays an important role in gonadal-intestinal axis homeostasis in female animals.

Threonine is a non-essential amino acid, while N-Stearoyl threonine is a lipoamino acids (LAs) formed by combining threonine with fatty acids. Previous research has shown that LAs, including N-Stearoyl Threonine, have neuroprotective properties possibly due to the presence of the fatty acyl group and the carboxyl and hydroxyl groups on the amino acid side chain (32). Studies have shown that the glycine-serine-threonine metabolic axis is a key metabolic center related to aging and longevity, and many related pathways are provided by mitochondria to provide energy in the form of ATP (33). In the aging rat model, anthocyanins improve aging and exert liver protection by regulating amino acid metabolic pathways such as L-Threonine (34). The ability of threonine to deliver glycine and acetyl-coa in mice via threonine dehydrogenase embodies an important link between cellular metabolism and epigenetically related pathways (35). The phosphorylation of threonine is crucial for intracellular signal transduction, regulating cell growth, differentiation, and apoptosis. L-threonine promotes the proliferation of mouse embryonic stem cells through lipid raft/microvesicle-dependent PI3K/Akt, MAPKs, and mTOR signaling pathways (36). It also enhances the phosphorylation of PI3K/Akt at Thr308 and Ser473 to activate other signaling cascades (36). Threonine effectively protects against cadmium-induced cellular apoptosis and membrane damage, and outperforms Vitamin C in restoring SOD activity *in vivo* (37). However, further investigation is required to fully understand the role of N-stearoyl threonine in regulating biological processes and ovarian function.

Ubiquinone, also known as coenzyme Q10 (CoQ10) in mammals, is a lipid-soluble antioxidant that is widely expressed in several organ systems (38). CoQ10 acts as an electron carrier to transfer electrons between mitochondrial respiratory chain enzyme complex I, II and III (39), thereby promoting the production of oxidative phosphorylated ATP to provide energy for cellular activities. CoQ10’s antioxidant effect can protect cell membranes from oxidative damage caused by free radicals (40). Female reproductive aging is often accompanied by oxidative stress and mitochondrial dysfunction, as confirmed by elevated ROS, decreased ATP levels and mitochondrial enzyme complex activity by ELISA validation in our study. The expression of CoQ10 synthesis genes, particularly Pdss2 and CoQ6, declines with age in both human and mouse oocytes (41). CoQ10 supplementation enhances oocyte quantity, glucose uptake, progesterone production (41), as well as mitochondrial activity and gene expression in oocytes (42). In elderly patients, ovarian granulosa cells exhibit a 50% decrease in activity of CoQ10-dependent mitochondrial respiratory chain enzyme complex III, indicating that CoQ10 deficiency is the underlying cause of mitochondrial dysfunction (39). Pretreatment with CoQ10 can enhance ovarian response, embryo quality and clinical pregnancy rate in women with DOR undergoing assisted reproductive cycles.

Integrating transcriptional and metabolomic data revealed that mitochondrial dysfunction is closely related to the pathogenesis of POI. Our previous transcriptome study showed that subunits of mitochondrial enzyme complex I, III, IV, and V were down-regulated in peripheral blood of POI women (8), and this phenotype was also confirmed by single-cell RNA-sequencing of aging mouse oocytes (43). Our study showed that CoQ10 expression was significantly reduced in POI, implying an imbalance of the antioxidant system and impaired mitochondrial energy metabolism in POI. Oxidative stress occurs when the accumulation of ROS exceeds the antioxidant defense system, inducing mitochondrial DNA mutations and leading to dysfunction (42). In fact, the electron transport system, enzyme complex activity, and oxidative phosphorylation are interconnected components of the energy metabolism within the mitochondrial respiratory chain. Any disruption in these processes can lead to mitochondrial dysfunction and impaired energy metabolism. Human oocytes possess a substantial number of mitochondria, and disturbances in mitochondrial function and energy metabolism can subsequently trigger granulosa cell apoptosis and follicle atresia, ultimately resulting in ovarian failure (**Figure 7**).

Although this study provides new insights into the metabolic features and molecular regulatory mechanisms of POI, there are still some limitations. Firstly, it should be noted that this is a single-center study with a limited sample size, and the presence of clinical heterogeneity may introduce bias to the metabolic profile, thus limiting the generalizability of our findings. Therefore, future multi-center prospective studies with larger sample sizes are warranted to verify the reliability of metabolic markers. Secondly, the predictive model was derived from single-center data, requiring recalibration when applied in other institutions. Utilizing multi-center large sample data can optimize the diagnostic model. Finally, this study exclusively focused on peripheral blood metabolomics; however, future investigations should encompass ovarian tissue, granulosa cells, and oocytes to elucidate the expression patterns and regulatory mechanisms of metabolic markers across diverse samples.

## 5 Conclusion

This non-target metabolomics study provided a panorama of changes in the plasma metabolic profile of POI. By employing bioinformatics, WGCNA and ML, we successfully identified eight metabolic markers and developed an XGBoost diagnostic model. The downregulation of these metabolic markers in POI may contribute to elevated oxidative stress levels and impaired energy metabolism. Furthermore, our integrated transcriptome and metabolomics data revealed that the decreased expression of mitochondrial respiratory chain electron carrier (CoQ10) and enzyme complex subunits led to inhibition of enzyme complex activity as well as disruption in oxidative phosphorylation process, ultimately resulting in reduced ATP production. Therefore, we speculated that the pathogenesis of POI is intricately linked to oxidative stress, MRC dysfunction, and energy metabolism disruption. These findings provide valuable insights into the pathological mechanisms of POI at both transcriptional and metabolic levels. Moreover, the identification of metabolites and prediction models holds significant implications for the diagnosis, treatment, and monitoring of POI.

## Data availability statement

The datasets presented in this study can be found in online repositories. The names of the repository/repositories and accession number(s) can be found in the article/**Supplementary Material**.

## Ethics statement

The studies involving humans were approved by the Ethics Committee of the First Affiliated Hospital of Guangxi Medical University (NO.2021KY-E-249). The studies were conducted in accordance with the local legislation and institutional requirements. The participants provided their written informed consent to participate in this study.

## Author contributions

ZY: Data curation, Validation, Visualization, Writing – original draft, Formal analysis, Software. HW: Conceptualization, Data curation, Validation, Writing – review & editing. ML: Project administration, Resources, Supervision, Conceptualization. XF: Conceptualization, Project administration. FL: Methodology, Software, Validation. JW: Data curation, Visualization. WP: Data curation, Methodology, Software, Investigation. HD: Data curation, Formal analysis.
