# Supplementary figures and images for: Integrated metabolomics and transcriptomics to reveal biomarkers and mitochondrial metabolic dysregulation of premature ovarian insufficiency
Source: Front Endocrinol (Lausanne). 2023 Dec 21;14:1280248. doi: 10.3389/fendo.2023.1280248 (PMC10764474; doi:10.3389/fendo.2023.1280248)

# Sample clustering to detect outliers

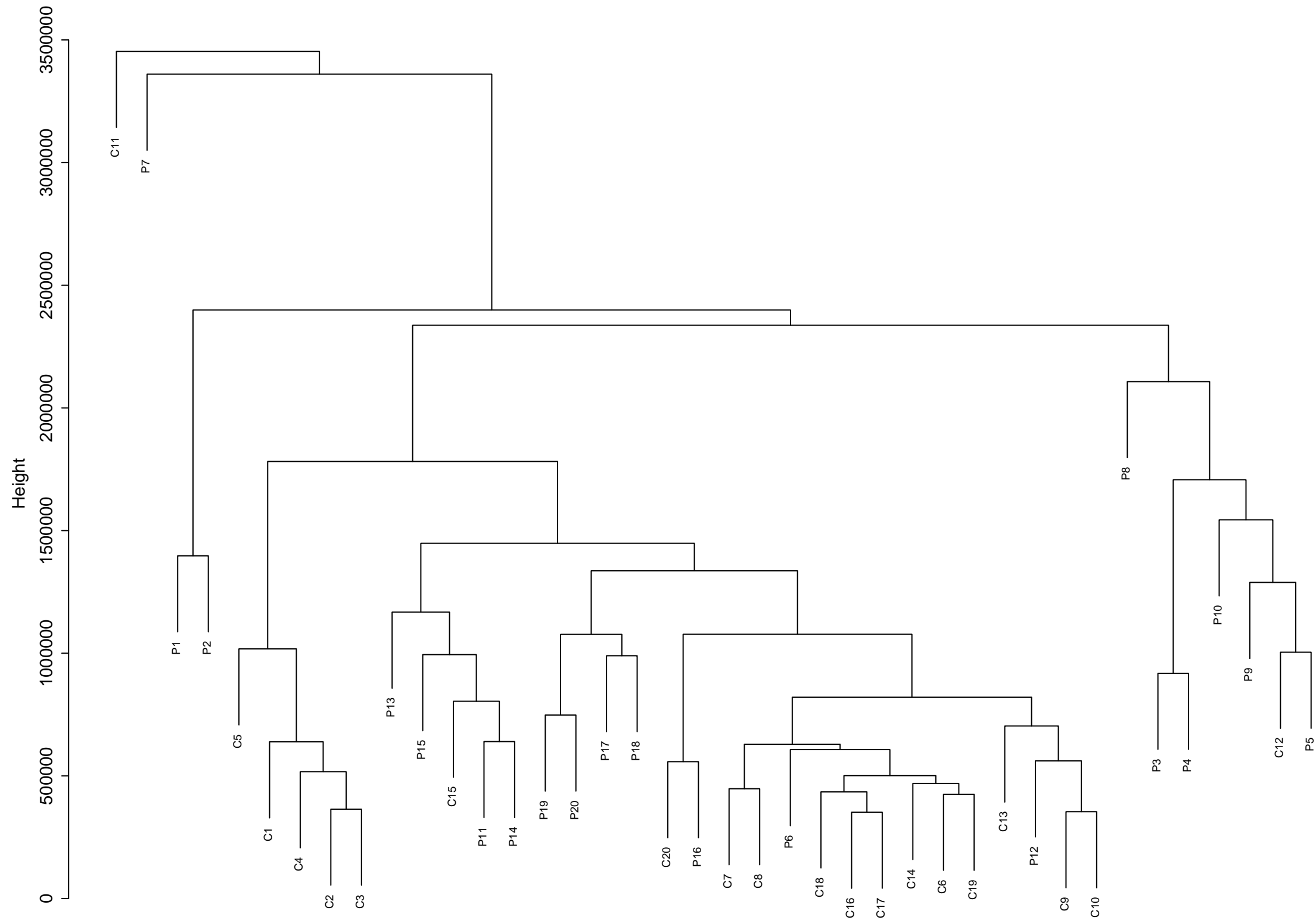

Supplement: Supplementary file 1 [file DataSheet_1.pdf]

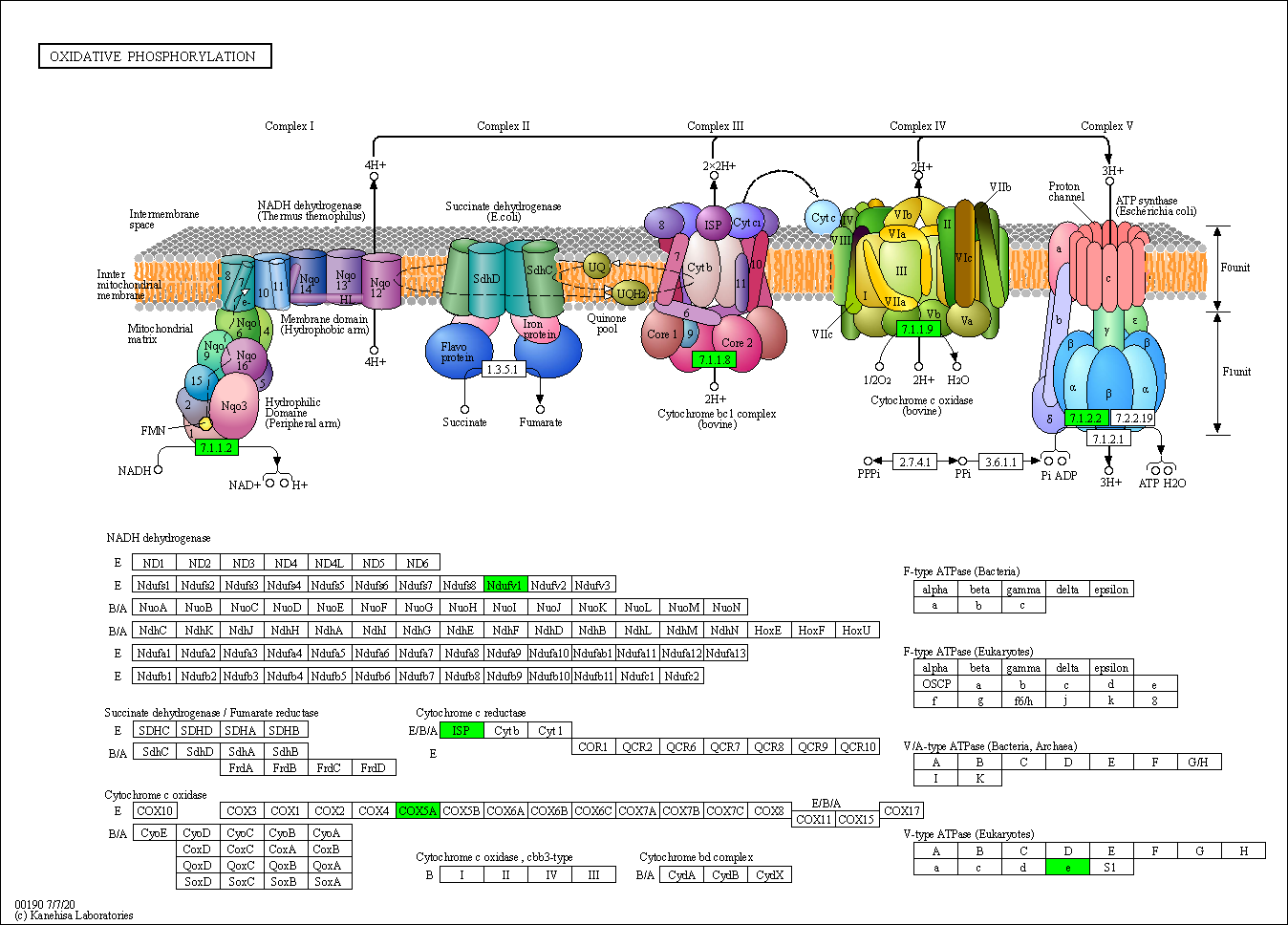

Supplement: Supplementary file 2 [file Image_1.png]
