# Supplementary material for: Integrated metabolomics and transcriptomics to reveal biomarkers and mitochondrial metabolic dysregulation of premature ovarian insufficiency
Source: Front Endocrinol (Lausanne). 2023 Dec 21;14:1280248. doi: 10.3389/fendo.2023.1280248 (PMC10764474; doi:10.3389/fendo.2023.1280248)
Supplement: Supplementary file 6 [file Table_4.docx]

**Supplementary Table 3 ROC curves and Youden index for metabolic markers**

| Variable | N | AUC | Sensitivity | Specificity | Youden index | Optimal threshold value |
| --- | --- | --- | --- | --- | --- | --- |
| RetinolVitaminA | 40 | 0.875 | 0.75 | 0.95 | 0.7 | 4456.691 |
| Ubiquinone | 40 | 0.89 | 0.8 | 0.9 | 0.7 | 113.884 |
| T1616dimethylPGA1 | 40 | 0.893 | 0.8 | 0.9 | 0.7 | 2512.501 |
| T15SHpEDE | 40 | 0.875 | 0.8 | 0.9 | 0.7 | 4568.063 |
| PA1302055Z8Z11Z14Z16EOH18R | 40 | 0.875 | 0.8 | 0.9 | 0.7 | 520.556 |
| T812Octadecadiynoicacid | 40 | 0.903 | 0.75 | 1.0 | 0.75 | 1998.024 |
| NStearoylThreonine | 40 | 0.848 | 0.75 | 0.9 | 0.65 | 1135.27 |
| T11deoxy1616dimethylPGE2 | 40 | 0.835 | 0.85 | 0.8 | 0.65 | 99.098 |
